# Pleomorphic Adenoma of Superficial and Deep Parotid Gland: A Case Report

**DOI:** 10.7759/cureus.62791

**Published:** 2024-06-20

**Authors:** Kesav Sudabattula, Anup Zade, Darshana Tote, Srinivasa Reddy, Tejaswini Panchagnula, Tushar Dahmiwal

**Affiliations:** 1 General Surgery, Jawaharlal Nehru Medical College, Datta Meghe Institute of Higher Education and Research, Wardha, IND; 2 Surgery, Mahatma Gandhi Institute of Medical Sciences, Wardha, IND; 3 Surgery, Jawaharlal Nehru Medical College, Datta Meghe Institute of Higher Education and Research, Wardha, IND; 4 General Surgery, Sapthagiri Institute of Medical Sciences and Research Centre, Benguluru, IND

**Keywords:** salivary gland tumors, pleomorphic adenoma, parotid gland, parotidectomy, benign mixed tumor

## Abstract

Parotid gland is the largest salivary gland of the body. Pleomorphic adenomas are the most prevalent benign parotid gland tumors. They can eventually grow to a size where they weigh several kilograms if not timely addressed. The ‘pleomorphic’ characteristics are attributed to the origin of the tumor from the connective tissue and epithelium. Pleomorphic adenomas often arise from the superficial lobe, further extending into the parapharyngeal space and gland’s other deeper tissues. Common incidence is noted in females between 30 and 50 years. Tumors typically present as asymptomatic swelling and progress slowly. The cornerstone of treatment is surgical removal of the tumor mass, with great care being given to protect the facial nerve. Most of these tumors are observed with the involvement of the superficial lobe; only a few are observed involving the deep lobe. This case report presents an intriguing case of a pleomorphic adenoma of superficial and deep parotid gland in a 65-year-old male. The left side of the patient's face had a steadily increasing, asymptomatic swelling on admission. Magnetic resonance imaging of the neck revealed a pleomorphic adenoma of the superficial and deep parotid gland. The patient underwent surgical excision of the parotid gland, which was uneventful.

## Introduction

Pleomorphic adenoma or benign mixed tumors are the common tumors of the salivary gland, which are reported to account for a total of two-thirds of all the salivary gland neoplasms, 60-80% benign salivary gland tumors, and 60-70% of all the parotid tumors [1,2]. The World Health Organization has defined more than 30 different subtypes of parotid tumors based on histological characteristics such as pleomorphic adenoma, lymphomatous papillary cystadenomas, mucoepidermoid carcinoma, adenoid cystic carcinoma, acinic cell carcinoma, and polymorphous low-grade adenocarcinoma [3]. These adenomas are most commonly observed in parotid glands, minor salivary glands, and submandibular glands at an incidence rate of 85%, 10%, and 5%, respectively. It majorly affects females aged between 30 and 50 years [4]. Pleomorphic adenoma presents as an irregular, rubbery, lobulated, slow-growing mass without any associated pain or discomfort. There is a small risk of malignant transformation into a carcinoma ex-pleomorphic adenoma, which is proportional to the time the lesion is in situ (1.5% in the first five years, 9.5% after 15 years). It is commonly observed in soft palate, hard palate, lips, tongue, cheek, and floor of the mouth [5]. There are two main surgical approaches: an enucleation (local dissection) sub-total superficial parotidectomy and total parotidectomy [6]. Surgical removal is carried out on the location of the tumor, which is either superficial parotidectomy or total parotidectomy for deep lobe tumors [2,6]. This is a case of a 65-year-old male who presented to the outpatient department of our hospital with an asymptomatic swelling in the left side of his face for the past 15 years with a slow progression in size.

## Case presentation

**Figure 1:**
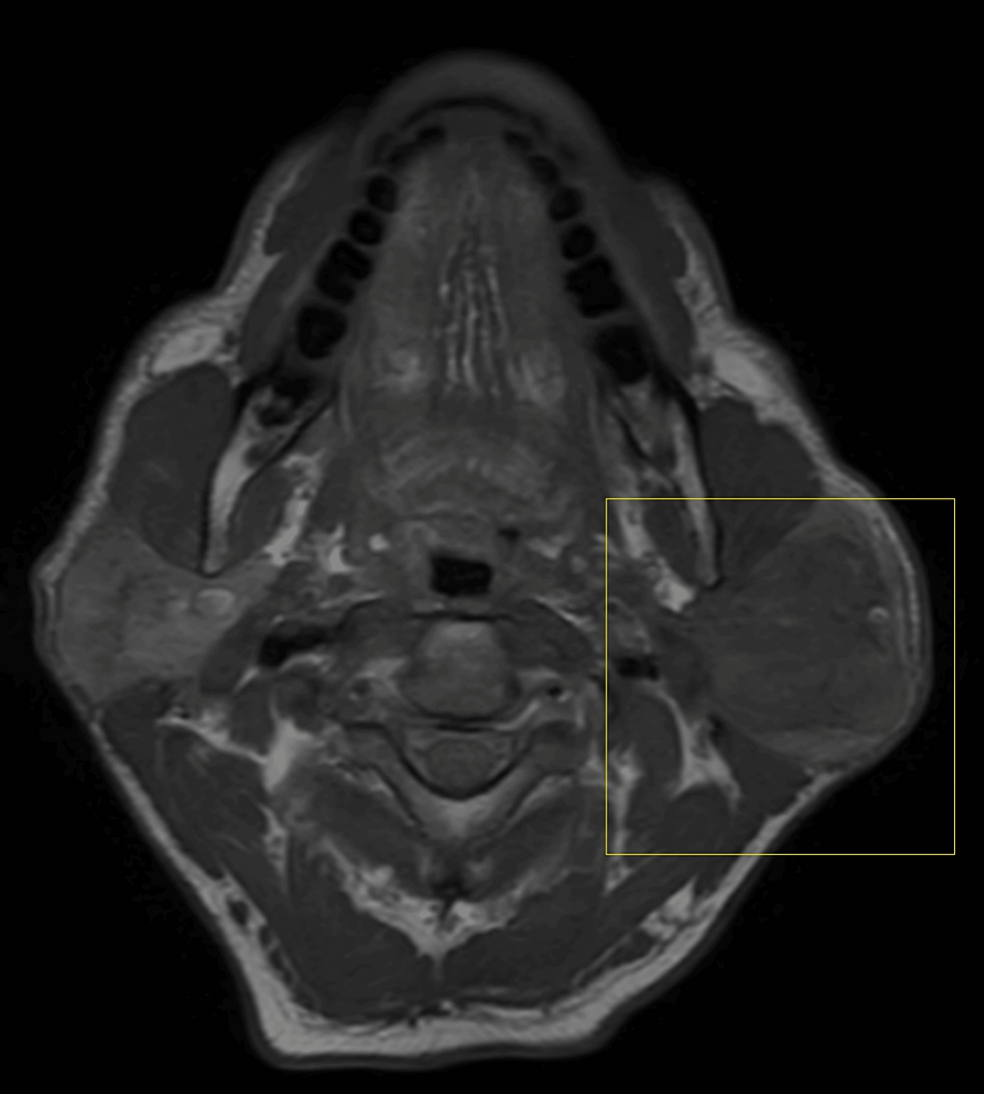
TIWI axial MRI image The highlighted area shows the physical presentation of parotid gland tumor T1WI, T1-weighted image

**Figure 2:**
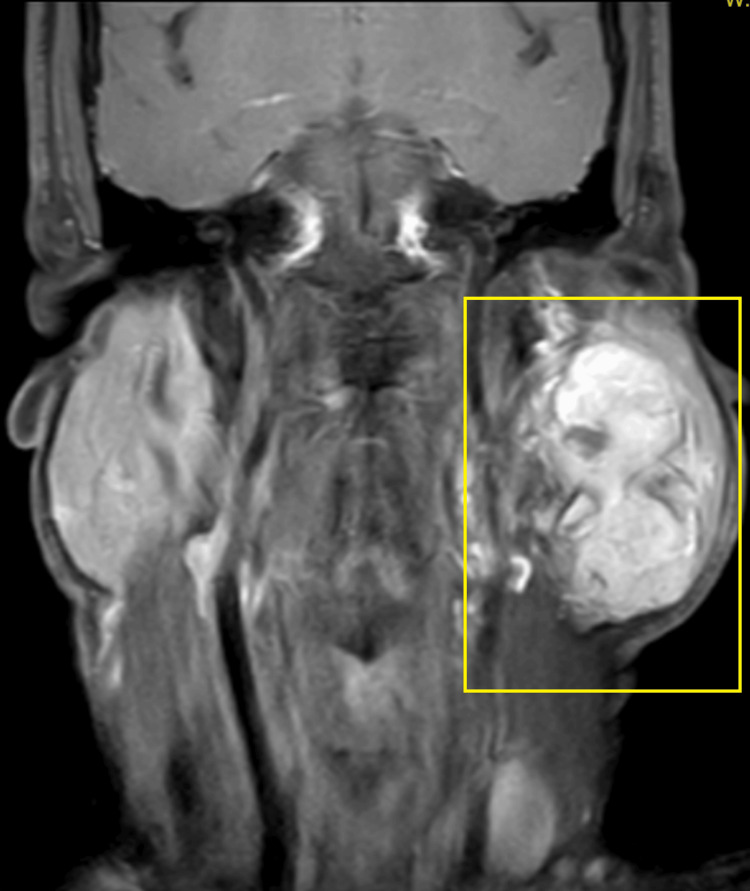
T1WI post-contrast coronal MRI image Highlighted area shows the physical presentation of parotid gland tumor T1WI, T1-weighted image

**Figure 3:**
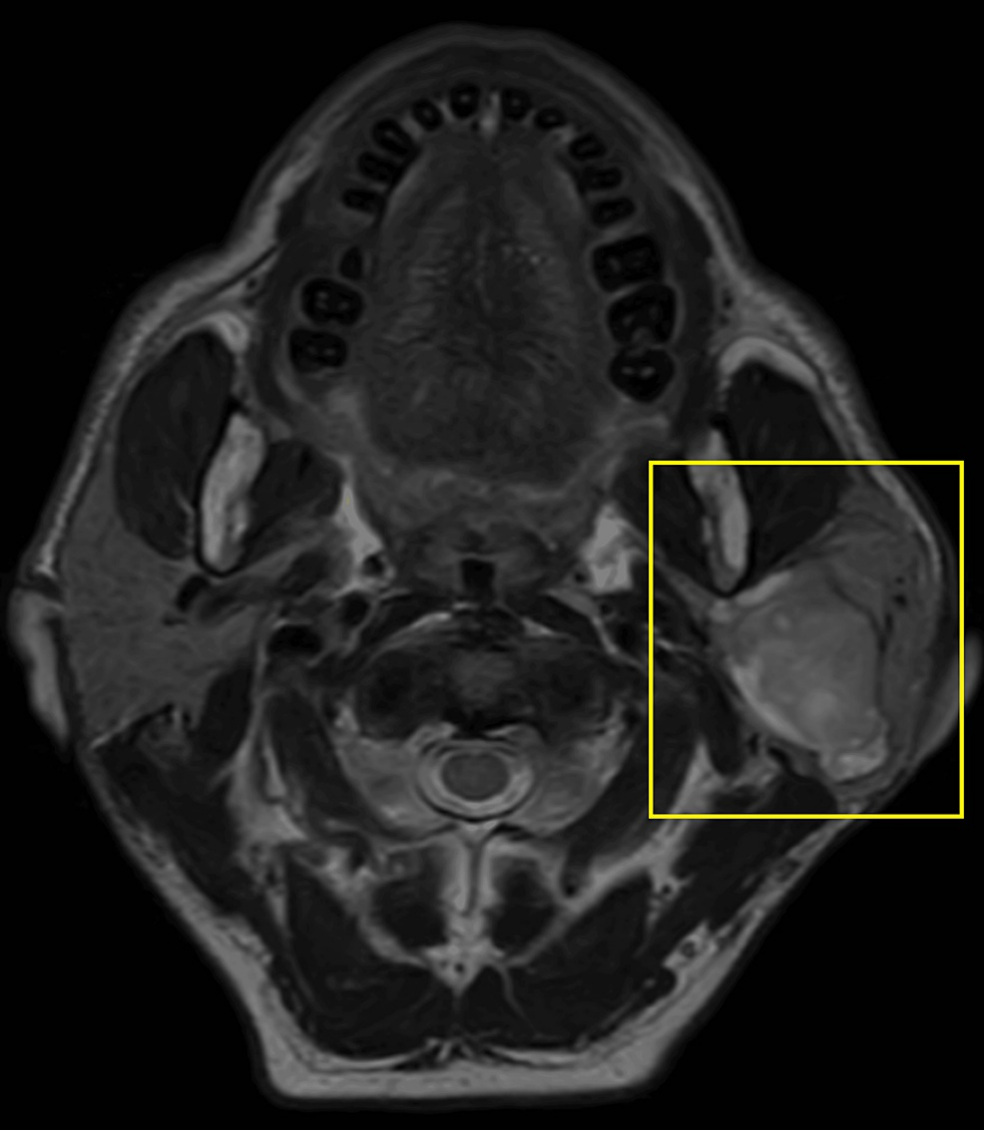
T2WI axial MRI image Highlighted area shows the physical presentation of parotid gland tumor T2WI, T2-weighted image

**Figure 4:**
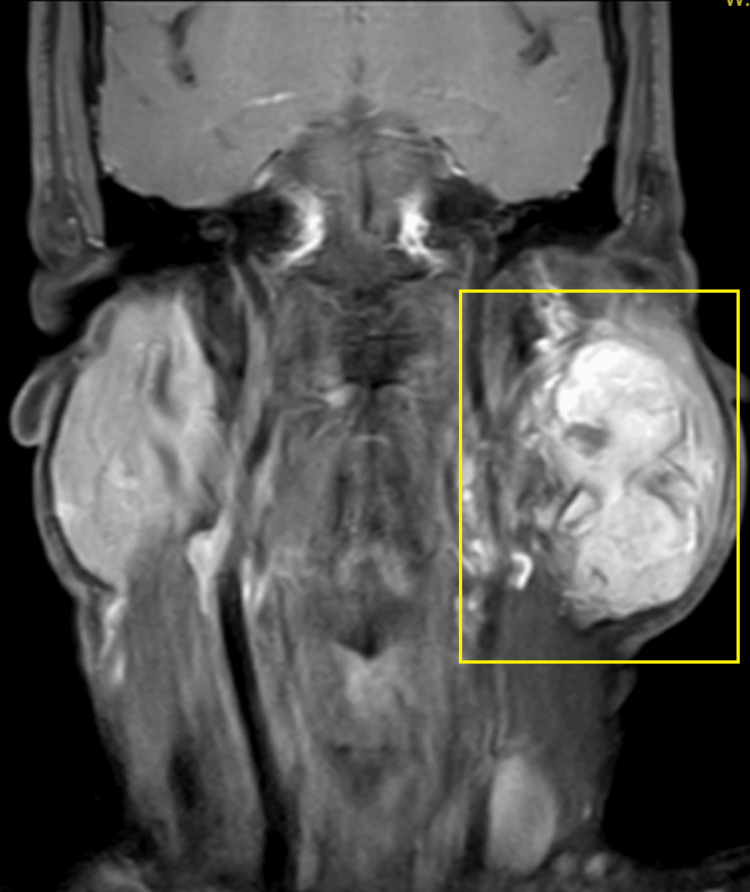
T1WI post-contrast sagittal MRI image Highlighted area showing the physical presentation of parotid gland tumor T1WI, T1-weighted image

A 65-year-old male visited the outpatient department with a complaint of swelling on the left side of his face. The swelling was observed to be progressive in nature, but asymptomatic and painless for the last 15 years. Initially, there was no significant growth in the swelling, but it grew to its current dimensions (7 x 9 cm) over a period of time. The patient has no significant medical history. On local extra-oral clinical examination, there was a marked facial asymmetry. An extra-oral, well-defined, solitary swelling was appreciated extending from the Ala-Tragus line superiorly up to approximately 2 cm below the mandibular angle inferiorly, and from the vertical lateral canthal line anteriorly up to the mastoid process posteriorly measuring approximately 7 x 9 cm with everted ear lobe and the swelling was firm, non-fluctuant, non-tender, and movable with no symptomatic involvement of the facial nerve. Ultrasound imaging of the left parotid gland was suggestive of a well-defined, lobulated heterogeneously hypoechoic lesion in the left angle of the mandible region. Color Doppler findings revealed a mass measuring 7 x 9 cm with minimal vascularity and parotid gland was not separately observed. Furthermore, magnetic resonance imaging (MRI) revealed a well-defined, heterogeneously enhancing large, lobulated, altered signal intensity lesion with few non-enhanced areas observed. The mass was noted involving both superficial and deep lobes of left parotid gland, appearing isotense on T1-weighted image (T1WI), heterogeneously hyper-intense on T2-weighted image (T2WI) measuring 5 x 5 x 8 cm and found to be displacing the left retromandibular vein laterally and a necrotic intra-parotid lymph node of size 13 x 12 mm was noted (Figures 1-4).

**Figure 5:**
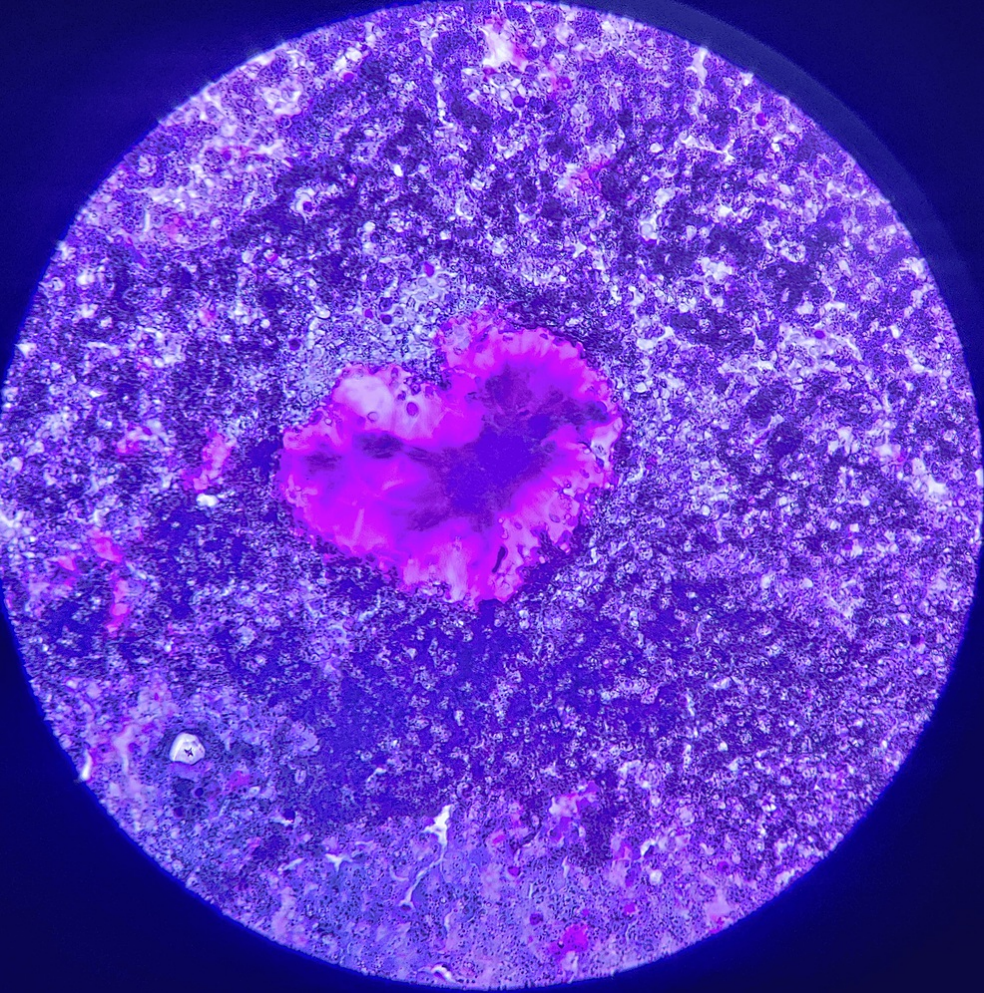
May Grunwald Giemsa stained slide of the specimen

A fine-needle aspiration cytology examination was suggestive of pleomorphic adenoma. The characteristic features were observed as cells arranged in single-layered sheets. These cells contain a moderate amount of cytoplasm with central nuclei surrounded by magenta-colored myxoid matrix fragments on May Grunwald Giemsa staining (Figure 5).

**Figure 6:**
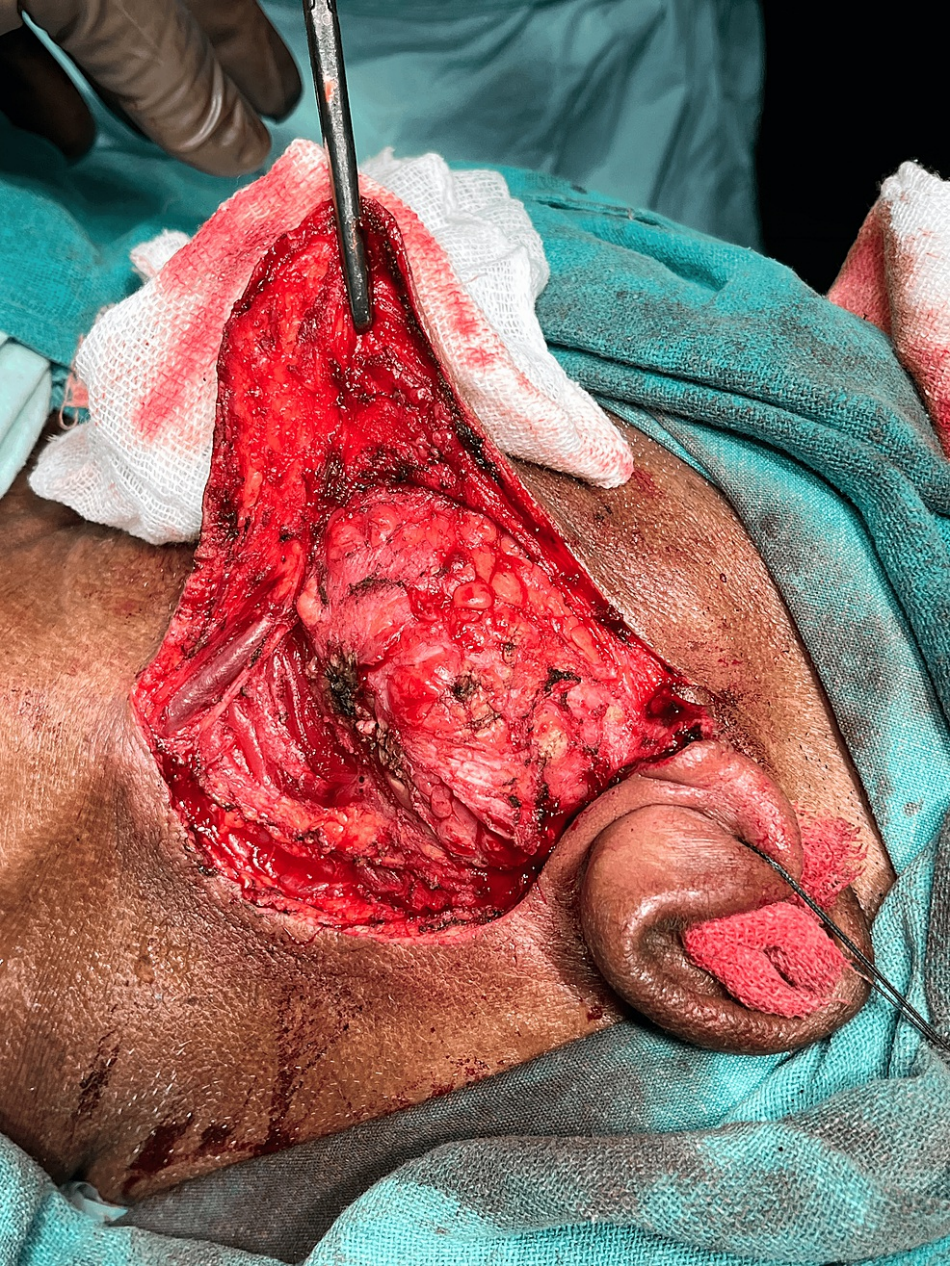
Pleomorphic adenoma of the parotid gland

**Figure 7:**
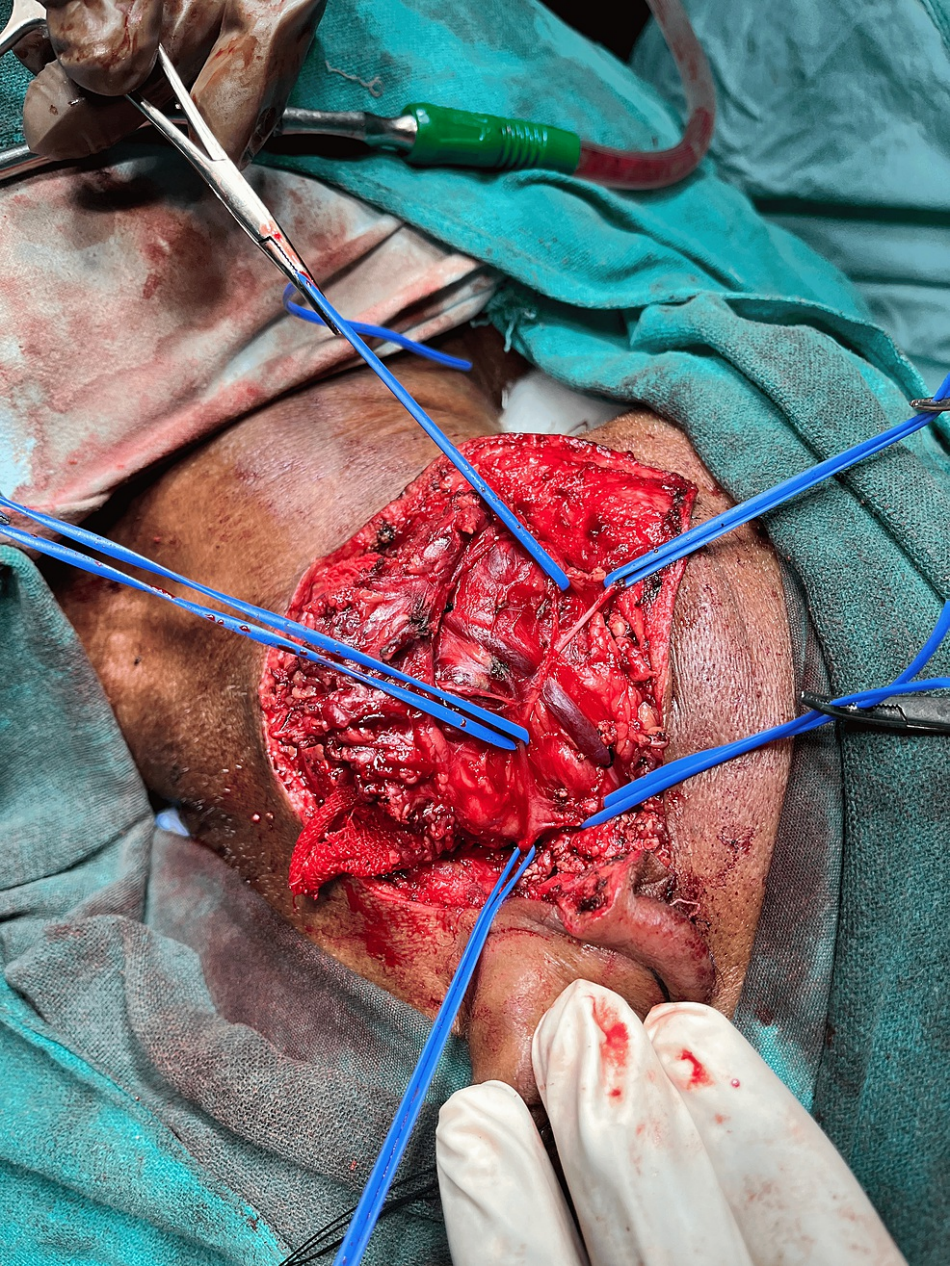
Facial nerve and its course

**Figure 8:**
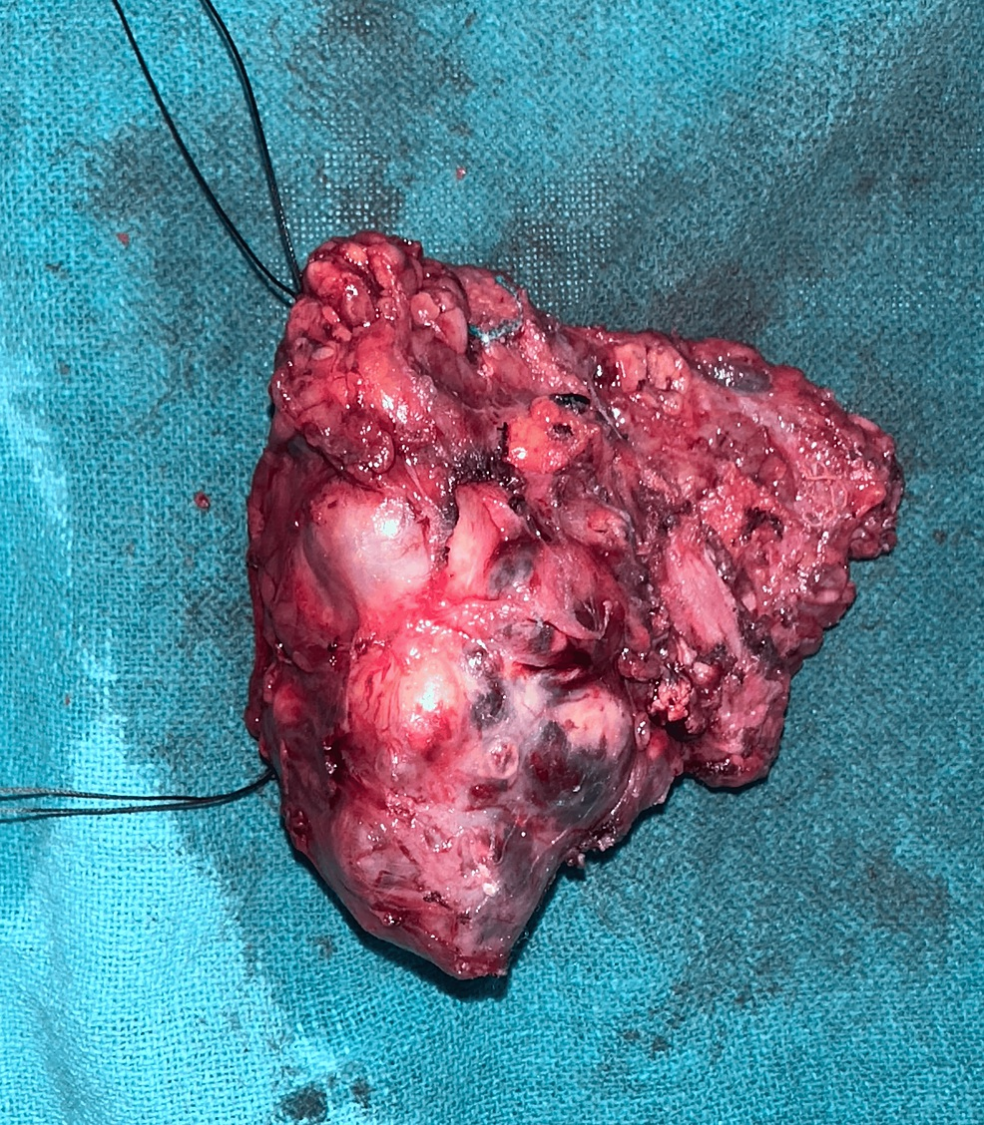
Excised specimen of parotid gland along with adenoma

**Figure 9:**
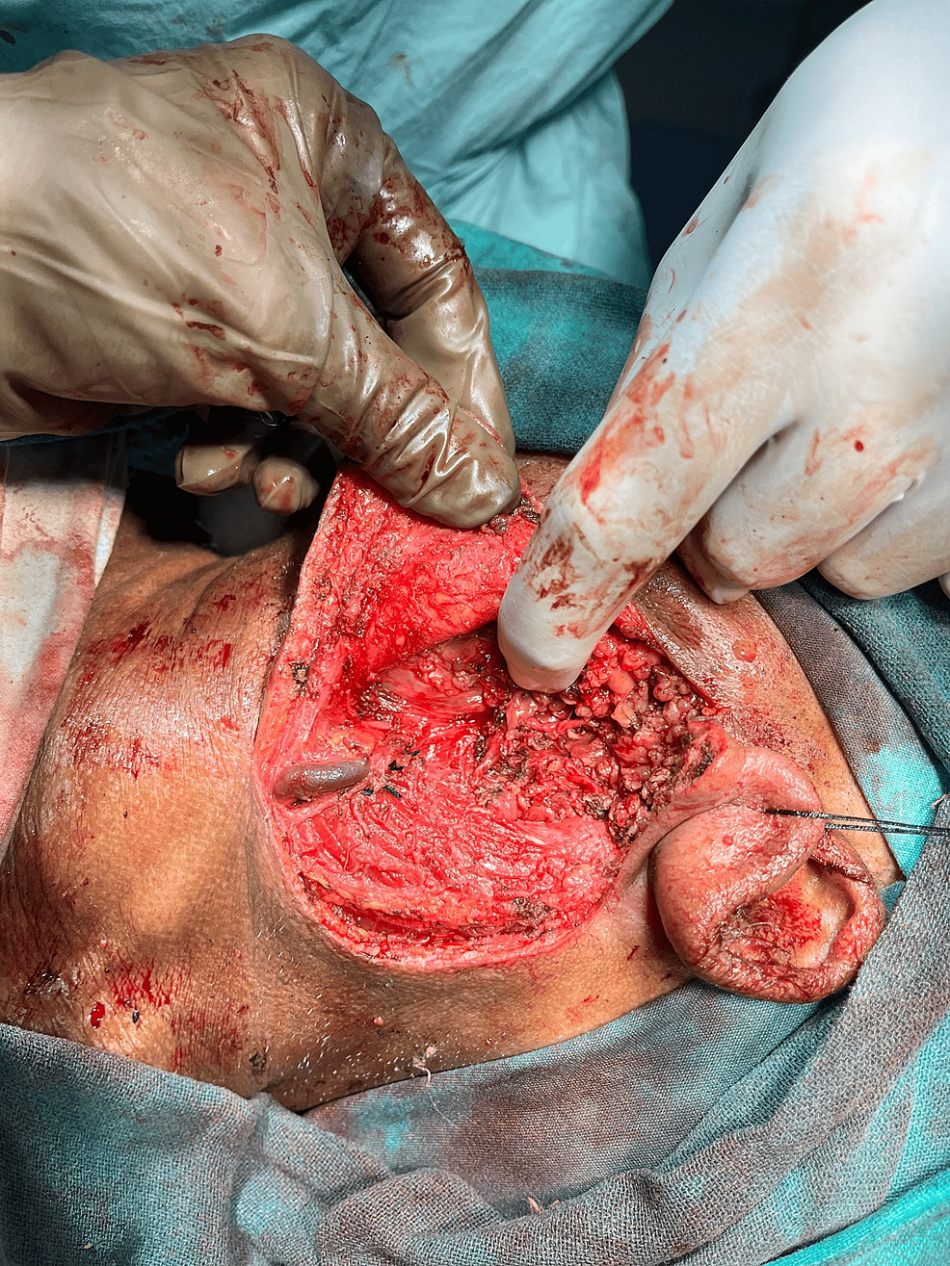
Post-excision image after deep lobe excision

The patient underwent total parotidectomy. Modified Blair incision was taken at the left pre-auricular region and nerves were separated from the gland and tumor along with the parotid gland, which was resected after separation followed by skin closure (Figures 6-9).

Post-operative recovery was good and uneventful, with intact facial nerve function. The patient was discharged after 10 days post removal of the sutures and advised to come for regular follow-up.

## Discussion

Pleomorphic adenomas are reported to occur at any age but are commonly observed in adults aged between 30 and 60 years [7]. There are multiple theories put forward regarding the histogenesis of these adenomas, though their etiology still remains unclear [1,4]. Some reports mentioned radiation exposure as a risk factor in increasing the incidence contrary to the theory that proposed simian virus 40 (SV40), genetic predisposition, chemical exposure, and tobacco usage as the reasons for their incidence [4,7,8]. Pleomorphic adenomas are observed both in epithelial and mesenchymal cells, which on normal physical examination can be noticed as an asymptomatic lump or swelling. These adenomas typically manifest as a swelling or lump for an extended period with progressive growth and be non-symptomatic even in cases where it grows in the vicinity of important parapharyngeal structures like nerves or veins or displace them [9]. 

Diagnostic modalities include an MRI or computed tomography (CT) scan. MRI has been found to be comparatively better in defining soft tissues and providing accurate information about the location of tumor margins and their relationship to the surrounding structures compared to CT imaging and can be preferred for pleomorphic adenoma [8]. MRI can be helpful in differentiating the tumor and its surrounding tissue involvement, which can further aid the surgical excision as in our case, where both superficial and deep lobe tumors were present. Another straightforward and trustworthy technique for the diagnosis of a salivary gland tumor is fine-needle aspiration cytology (FNAC). There are reports indicating that FNAC has a sensitivity of over 98% and a specificity of roughly 85% to 95%. Misdiagnosis can lead to incorrect tumor classification, but it is uncommon for a benign FNA tumor to be mistakenly identified as a malignant neoplasm [10]. Currently, there are two treatment options for pleomorphic adenoma of the parotid gland: superficial (Patey's operation) or total parotidectomy. The latter is preferred because of its lower recurrence rate, as done in our case [10,11]. 

The treatment for submandibular gland tumors involves a straightforward excision surgery, which preserves nearby nerves, such as the lingual, hypoglossal, and mandibular branches of the trigeminal nerve [11,12]. An enucleation procedure was also used for the treatment of pleomorphic adenoma, but it is not recommended at present due to its high recurrence rates [6]. A 5-mm margin is recommended for the minor salivary gland tumors as the periosteum is not invaded by these malignancies, hence resection of the bone is not needed. Tumor bed recurrences exhibit substantial resistance to treatment, and the only available alternatives for management are radiation therapy, surgery, and monitoring alone. There is a slight chance of pleuromorphic adenomas getting converted into malignant tumors. The length of time the lesion is present determines the likelihood of malignancy (1.5% during the first five years, 9.5% after 15 years), which results in the absolute necessity of an excision. Radiation therapy, large size, recurring tumors, and advanced age are additional risk factors for malignancy [13].

## Conclusions

Pleomorphic adenomas are prone to recurrence and malignant change; therefore, they require careful management. Facial nerve protection is the primary focus in the surgical management of the tumors of the deep lobe of the parotid gland. The last course of treatment is conservative total parotidectomy because enucleation may lead to recurrence. A long-term follow-up is required to look for recurrence even after the removal of adenoma. Throughout the course of the follow-up, the patient needs to be closely monitored for any indications of recurrence.
